# Association of Exposure to Endocrine-Disrupting Chemicals During Adolescence With Attention-Deficit/Hyperactivity Disorder–Related Behaviors

**DOI:** 10.1001/jamanetworkopen.2020.15041

**Published:** 2020-08-28

**Authors:** Jessica R. Shoaff, Brent Coull, Jennifer Weuve, David C. Bellinger, Antonia M. Calafat, Susan L. Schantz, Susan A. Korrick

**Affiliations:** 1 Channing Division of Network Medicine, Harvard Medical School, Brigham and Women’s Hospital, Boston, Massachusetts; 2 Department of Epidemiology, Harvard T.H. Chan School of Public Health, Boston, Massachusetts; 3 Department of Biostatistics, Harvard T.H. Chan School of Public Health, Boston, Massachusetts; 4 Department of Environmental Health, Harvard T.H. Chan School of Public Health, Boston, Massachusetts; 5 Department of Epidemiology, Boston University School of Public Health, Boston, Massachusetts; 6 Department of Neurology and Psychiatry, Boston Children’s Hospital and Harvard Medical School, Boston, Massachusetts; 7 Division of Laboratory Sciences, National Center for Environmental Health, Centers for Disease Control and Prevention, Atlanta, Georgia; 8 Beckman Institute for Advanced Science and Technology, University of Illinois at Urbana-Champaign, Urbana; 9 Department of Comparative Biosciences, University of Illinois at Urbana-Champaign, Urbana

## Abstract

**Importance:**

Attention-deficit/hyperactivity disorder (ADHD) is the most common childhood neurobehavioral disorder. Studies suggest that prenatal and early childhood exposure to endocrine-disrupting chemicals may be associated with ADHD, but the association during adolescence has not been studied to date.

**Objective:**

To evaluate the association between exposure to select endocrine-disrupting chemicals during adolescence and ADHD-related behaviors.

**Design, Setting, and Participants:**

For this cross-sectional analysis, data were collected from 205 adolescents in the New Bedford Cohort, an ongoing prospective birth cohort, between June 18, 2011, and June 10, 2014. The adolescents provided spot urine samples and underwent neurodevelopmental testing. Statistical analyses performed from January 15 to December 31, 2019, used a repeated-measures analysis with multivariate modified Poisson models to estimate the adjusted relative risk of ADHD-related behaviors associated with exposure to endocrine-disrupting chemicals.

**Exposures:**

Urinary biomarker concentrations of endocrine-disrupting chemicals or their metabolites, including phthalates, parabens, phenols, and triclocarban, were quantified. Summary exposure measures were created, combining biomarker concentrations of chemicals with a shared mechanism of action, exposure pathway, or chemical class.

**Main Outcomes and Measures:**

Behaviors related to ADHD were assessed with up to 14 indices from self-, parent-, and teacher-completed behavioral checklists using validated and standardized instruments; specifically, the Conners Attention Deficit Scale and the Behavior Assessment System for Children, Second Edition. Scores on each index were dichotomized to identify those with evidence of a significant behavioral problem, defined by each scale’s interpretive guidelines.

**Results:**

Among the 205 participants, the mean (SD) age at assessment was 15.3 (0.7) years, with 112 girls (55%) and 124 non-Hispanic White participants (61%). The median urine concentrations were 0.45 μmol/L of Σantiandrogenic phthalates, 0.13 μmol/L of ΣDEHP metabolites, 0.49 μmol/L of Σpersonal care product phthalates, 0.35 μmol/L of Σparabens, 0.02 μmol/L of Σbisphenols, and 0.02 μmol/L of Σdichlorophenols. A total of 82 (40%) had scores consistent with a significant behavioral problem, whereas 39 (19%) had an ADHD diagnosis. Each 2-fold increase in the sum of antiandrogenic phthalate concentrations was associated with a 1.34 (95% CI, 1.00-1.79) increase in the risk of significant ADHD-related behavior problems, whereas a 2-fold increase in the sum of dichlorophenols was associated with a 1.15 (95% CI, 1.01-1.32) increased risk. These associations tended to be stronger in male participants, but comparisons of sex-specific differences were imprecise.

**Conclusions and Relevance:**

Endocrine-disrupting chemicals are used in a wide variety of consumer products resulting in ubiquitous exposure. The study findings suggest that exposure to some of these chemicals, particularly certain phthalates, during adolescence may be associated with behaviors characteristic of ADHD.

## Key Points

**Question:**

Is exposure to endocrine-disrupting chemicals during adolescence a risk factor for behaviors associated with attention-deficit/hyperactivity disorder (ADHD)?

**Findings:**

In this cross-sectional analysis of data from 205 adolescents participating in a prospective birth cohort study, exposure to select phthalates was associated with an increased risk of ADHD-related behaviors. Associations were strongest for phthalates with antiandrogenic activity.

**Meaning:**

These findings support the potential importance of exposures to endocrine-disrupting chemicals, especially phthalates, during adolescence as correlates of ADHD-related behaviors.

## Teaser

This cross-sectional analysis evaluates the association between exposure to select endocrine-disrupting chemicals during adolescence and attention-deficit/hyperactivity (ADHD)–related behaviors.

## Introduction

Attention-deficit/hyperactivity disorder (ADHD) is the most common neurobehavioral disorder of childhood, affecting approximately 9.4% of children in the United States.^1^ Attention-deficit/hyperactivity disorder is characterized by difficulty maintaining attention, controlling impulses, and regulating activity level and may be associated with academic and social problems as well as difficulties throughout adulthood.^2,3,4,5^

There is growing evidence that exposure to endocrine-disrupting chemicals (EDCs), such as phthalates and phenols, may be associated with ADHD.^6,7,8,9^ Phthalates and phenols are widely used in consumer products, including food processing and packaging equipment, personal care products (eg, cosmetics and fragrances), and pharmaceuticals, resulting in ubiquitous exposure.^6,10,11^ Although epidemiologic studies have reported associations between prenatal and early childhood exposure to EDCs and ADHD-like behaviors,^8,12,13^ few studies have examined the association of exposure to these chemicals during adolescence with ADHD-like behaviors. Similar to the prenatal period, adolescence is a critical time for brain development, characterized by structural and functional changes in the brain as well as the onset of behavioral problems, some of which may be due to hormonal changes.^14^ As such, exposure to EDCs during adolescence may be particularly detrimental.

The objective of this study is to examine the association of adolescent exposure to EDCs, specifically phthalates, phenols, and triclocarban, with ADHD-related behaviors. We hypothesize that exposure to certain EDCs during adolescence is particularly detrimental to adolescent behavior given the rapid brain development that occurs during this time.

## Methods

### Study Population

We analyzed data from the New Bedford Cohort, a prospective birth cohort of mother-infant pairs recruited after delivery at a New Bedford, Massachusetts, hospital between 1993 and 1998.^15^ The original aim of the study was to examine the association of prenatal organochlorine and metal exposures with subsequent neurodevelopment among children living near the New Bedford Harbor Superfund site. Eligibility requirements included newborns who were vaginally delivered to mothers who were at least 18 years of age and who were available for neonatal examination. In-person neurodevelopmental testing was performed when participants were approximately 15 years of age between 2008 and 2014. Of the 788 newborns enrolled in the New Bedford Cohort, 660 (84%) met eligibility criteria (residence in the study region, absence of catastrophic central nervous system injury or disease, available biomarkers of early chemical exposure, and contact information) for these assessments and, of those newborns, 528 (80%) completed assessments. Midway through the 15-year follow-up, the study added assessment of exposure to EDCs. We invited the 252 participants who were evaluated in this last half of the study to provide spot urine samples at 2 points: during neurodevelopmental assessments and approximately 1 week later (mean duration of time after assessment, 7 days [range, 1-35 days]) (eFigure in the Supplement). This study followed the Strengthening the Reporting of Observational Studies in Epidemiology (STROBE) reporting guideline. The study research protocol was reviewed and approved by the human participants committee of the Brigham and Women’s Hospital (Boston, Massachusetts). Written informed parental consent and child assent were obtained from participants. The analysis of deidentified specimens at the Centers for Disease Control and Prevention (CDC) laboratory (Atlanta, Georgia) was determined not to constitute engagement in human participants research.

### Urine Sample Collection and EDC Measurement

Urine sample collection and analysis have been described elsewhere.^16^ In brief, of the 252 adolescents invited to provide urine samples, 205 (81%) provided at least 1 sample. Urine samples were collected in sterile polypropylene cups and frozen until processing and analysis.^16^ It was not possible to analyze all urine specimens, owing to resource limitations; of the 144 adolescents (57%) who provided 2 samples, 60 had each sample analyzed separately, and the mean concentration was used in analyses. For the remaining 84, equal volumes of the 2 samples were pooled for analysis.^16^ Specific gravity was measured using a digital refractometer (Pocket PAL-10S; ATACO USA Inc).

Urine concentrations of 28 biomarkers were quantified at the CDC in 2 batches (2012 and 2016), based on the timing of collection.^16,17,18,19,20^ Batch 1 included 11 phthalate metabolites and 8 phenols. Five additional biomarkers of phthalates or the phthalate substitute cyclohexane-1,2-dicarboxylic acid, monohydroxy isononyl ester (MHINCH), 3 additional phenols, and triclocarban were measured in batch 2 (Table 1).

**Table 1.** Distribution of Urinary Biomarker Concentrations of Phthalates, Phenols, Their Substitutes, and Triclocarban Among 205 Adolescent Participants in the New Bedford Cohort Who Provided Urine Samples From 2011 to 2014

Biomarker^a^	Full chemical name	5th Percentile	25th Percentile	50th Percentile	75th Percentile	95th Percentile
ΣAntiandrogen phthalates^b^^,^^c^	NA	0.11	0.26	0.45	0.71	1.41
ΣDEHP metabolites^b^	NA	0.03	0.08	0.13	0.19	0.50
ΣPersonal care product phthalates^b^	NA	0.09	0.24	0.49	0.97	3.22
ΣParabens^b^^,^^c^	NA	0.03	0.05	0.35	1.15	5.83
ΣBisphenols	NA	0.01	0.01	0.02	0.03	0.07
ΣDichlorophenols	NA	0.00	0.01	0.02	0.03	0.15
Individual chemicals						
MEP	Monoethyl phthalate	7.90	23.9	45.0	122	423
MBP	Mono-n-butyl phthalate	2.40	8.50	16.0	24.3	53.6
MiBP	Monoisobutyl phthalate	2.10	6.60	11.5	19.3	38.0
MHBP^c^	Monohydroxybutyl phthalate	0.20	0.70	1.50	2.85	6.60
MHiBP^c^	Monohydroxyisobutyl phthalate	1.10	2.30	4.00	7.30	17.5
MBzP	Monobenzyl phthalate	1.3	4.5	9.3	17.8	64.3
MEHP	Mono-2-ethylhexyl phthalate	0.10 (<LOD)	0.70	1.50	3.20	9.70
MEHHP	Mono-2-ethyl-5-hydroxyhexyl phthalate	2.20	6.20	10.5	17.4	45.4
MEOHP	Mono-2-ethyl-5-oxohexyl phthalate	1.60	4.80	7.70	11.7	33.7
MECPP	Mono-2-ethyl-5-carboxypentyl phthalate	5.20	11.60	18.5	28.1	70.8
MCOP	Monocarboxyoctyl phthalate	7.40	26.0	49.4	103.0	214.0
MNP^c^	Mono-isononyl phthalate	0.20	0.70	1.60	4.30	15.1
MCNP	Monocarboxynonyl phthalate	1.30	2.90	4.65	7.10	13.60
MCPP	Mono-3-carboxypropyl phthalate	0.9	2.5	5.0	10.2	43.6
MHINCH^c^^,^^d^	Cyclohexane-1,2-dicarboxylic acid, monohydroxy isononyl ester	0.00 (<LOD)	0.10	0.30	0.50	1.10
M-paraben	Methyl paraben	3.60	7.50	42.4	140	598
P-paraben	Propyl paraben	0.30	1.00	3.70	18.6	117
E-paraben^c^	Ethyl paraben	0.20	0.40	0.70	2.10	29.0
B-paraben	Butyl paraben	0.00 (<LOD)	0.00 (<LOD)	0.20	0.50	4.1
BPA	Bisphenol A	0.50	1.00	1.70	2.80	6.70
BPS^c^	Bisphenol S	0.10	0.20	0.40	0.80	2.30
BPF^c^	Bisphenol F	0.00 (<LOD)	0.10	0.20	0.60	5.50
BP-3	Benzophenone-3	4.15	13.0	38.3	172	1148
2,4-DCP	2,4-Dichlorophenol	0.15	0.40	0.60	1.10	2.30
2,5-DCP	2,5-Dichlorophenol	0.40	0.80	1.65	4.20	22.7
TCS	Triclosan	1.50	3.20	8.50	49.9	381
TCC	Triclocarban	0.00 (<LOD)	0.00 (<LOD)	0.10	0.20	4.70

Abbreviations: DEHP, di(2-ethylhexyl) phthalate; LOD, limit of detection; NA, not applicable; Σ, molar sum.

^a^ Units for sums are μmol/L and units for individual biomarkers are μg/L.

^b^ Concentrations were calculated as follows: Σantiandrogen phthalates (μmol/L): molar sum of MEHHP, MEHP, MEOHP, MECPP, MBP, MiBP, MBzP, MHiBP, MCOP, MNP, and MHBP (MCOP and MNP were downweighted by multiplying their molar concentrations by 0.43 prior to summing to reflect the potency of their parent compound relative to the other antiandrogenic phthalates); Σpersonal care product phthalates (μmol/L): molar sum of MBP, MHBP, MEP, MiBP, and MHiBP; ΣDEHP metabolites (μmol/L): molar sum of MECPP, MEHHP, MEOHP, MEHP; Σparabens (μmol/L): molar sum of B-paraben, E-paraben, M-paraben, and P-paraben; Σbisphenols (μmol/L): molar sum of BPA, BPF, and BPS; and Σdichlorophenols (μmol/L): molar sum of 2,4-dichlorophenol and 2,5-dichlorophenol.

^c^ Because some chemicals were not measured in the first batch of Centers for Disease Control and Prevention analyses, Σantiandrogen phthalates, Σpersonal care product phthalates, Σparabens, Σbisphenols, MHINCH, MCOCH, BPS, BPF, MNP, MHBP, MHiBP, TCC, and E-paraben concentrations were available for only 178 of the 205 participants.

^d^ Phthalate replacement.

Because batch 1 analyses did not include all biomarkers, urinary concentrations of all analytes were available for 178 participants (87%), with the remaining 27 participants missing concentrations of MHINCH, monohydroxyisobutyl phthalate (MHiBP), monoisononyl phthalate (MNP), monohydroxybutyl phthalate (MHBP), bisphenol F (BPF), bisphenol S (BPS), ethylparaben, and triclocarban. The limits of detection for the target analytes ranged from 0.2 to 2.3 μg/L; for concentrations less than the limit of detection, instrument readings were used in data analyses. The study-specific urine quality assurance and quality control samples for both between-batch and within-batch analyses demonstrated the excellent reproducibility of the analytic chemistry methods used.^16^

### Exposure Assessment

Because people are routinely exposed to multiple EDCs, we created summary exposure measures by combining biomarker concentrations for chemicals with a shared mechanism of action, exposure pathway, or chemical class. As described elsewhere,^16^ the molar sum (Σ) of 11 phthalate metabolites derived from antiandrogenic parent compounds was created to estimate Σantiandrogenic phthalates (in units of micromoles per liter): mono-2-ethyl-5-hydroxyhexyl phthalate (MEHHP), mono-2-ethylhexyl phthalate (MEHP), mono-2-ethyl-5-oxohexyl phthalate (MEOHP), mono-2-ethyl-5-carboxypentyl phthalate (MECPP), mono-n-butyl phthalate (MBP), monoisobutyl phthalate (MiBP), monobenzyl phthalate (MBzP), MHiBP, monocarboxyoctyl phthalate (MCOP), MNP, and MHBP.^16,21,22,23^ Summary measures for phthalates found in personal care products (Σpersonal care products; in units of micromoles per liter) was the molar sum of monoethyl phthalate (MEP), MBP, MHBP, MiBP, and MHiBP; the sum of di(2-ethylhexyl) phthalate (DEHP) metabolites (ΣDEHP; in units of micromoles per liter) was the molar sum of MECPP, MEHHP, MEOHP, and MEHP; total parabens (Σparabens; in units of micromoles per liter) was the molar sum of butyl, ethyl, methyl, and propyl paraben; total bisphenols (Σbisphenols; in units of micromoles per liter) was the molar sum of bisphenol A (BPA) and its replacements BPF and BPS; and total dichlorophenols (Σdichlorophenols; in units of micromoles per liter) was the molar sum of 2,4-dichlorophenol and 2,5-dichlorophenol.

### Behavior Assessment

Attention-deficit/hyperactivity disorder–related behaviors were assessed using validated behavioral checklists from the Behavior Assessment System for Children, Second Edition (BASC-2; parent-, teacher-, and self-reported) and Conners Attention Deficit Scale (CADS; parent- and teacher-reported).^24,25^ Both instruments have excellent internal reliability, with coefficient α values of 0.85 to 0.96 for the CADS and 0.74 to 0.95 for the BASC-2 (hyperactivity and attention problem scales), depending on the reporter. The BASC-2 scales are moderately correlated (*r* ∼ 0.5-0.6) with the Conners ADHD index, which, in turn, has excellent discriminant validity for ADHD diagnosis.^24,25^ Parents and adolescents completed these checklists at the study visit and first urine sample collection, while teachers completed them a median (SD) of 2.5 (6.6) months after the first urine sample collection. The BASC-2 consists of 139 to 176 questions, and the CADS consists of 14 to 15 questions, depending on the reporter. For each question, the frequency of adverse behavior is ranked using a 4-point Likert scale. Responses generate composite behavioral indices expressed as age- and sex-adjusted T-scores standardized to a mean (SD) of 50 (10), where a higher score indicates more adverse behavior. These measures generate 14 ADHD-related behavior indices. From the BASC-2, we included indices of inattention (teacher-, parent-, and self-reported), hyperactivity (teacher-, parent-, and self-reported), and executive function (teacher- and parent-reported). From the CADS parent and teacher reports, we included indices for inattention and hyperactivity based on *Diagnostic and Statistical Manual of Mental Disorders* (Fourth Edition) diagnostic criteria and a CADS index of overall ADHD behaviors. All 205 adolescents with exposure data had at least 1 outcome measure, 204 (99.5%) had parent- and self-completed checklists, and 173 (84%) had teacher-completed checklists.

### Covariates

Data on parental demographic charateristics (race/ethnicity, educational level, and income), health history, and tobacco use during pregnancy were collected in a questionnaire administered to mothers approximately 2 weeks after delivery. When the child was 15 years of age, the data on demographic characteristics, smoking habits, and child medical history (including behavioral disorder diagnoses and medication use) were updated via medical record reviews and questionnaires. Self-reported adolescent tobacco, alcohol, and marijuana use was ascertained with the CDC Youth Risk Behavior Survey. Umbilical cord serum levels of polychlorinated biphenyls and dichlorodiphenyldichloroethylene were used as biomarkers of prenatal organochlorine exposures. Childhood blood lead levels were abstracted from medical records of lead exposure screening; the maximum concentration between 12 and 36 months of age was calculated.

Using a structured diary, adolescents recorded food consumption and personal care product use for the 24 hours prior to urine sample collection. Diary data were used to estimate exposure risk factors during that 24-hour period, including the number of personal care products used and caffeinated beverages consumed, as well as the amount of fast food or canned food consumed. Height and weight were measured at the time of urine sample collection, and body mass index *z* scores were calculated using CDC US reference data.^26^

### Statistical Analysis

Statistical analyses were performed from January 15 to December 31, 2019. Regression diagnostics supported log_2_ transformation of urinary biomarker concentrations. We dichotomized outcomes at the 98th percentile, which BASC-2 and CADS guidelines define as indicative of significant behavioral problems. To leverage the multiple indices of ADHD-related behavior, maximize power, and avoid multiple comparisons, we used a repeated-measures analysis design, considering each of the 14 binary indices as a repeated measure reflecting a single underlying outcome.^27,28^ We used multivariate modified Poisson models with robust error variance and an independent working correlation that can accommodate missing behavioral scores, so participants with at least 1 behavioral measure were included. Analyses were performed using SAS, version 9.4 (SAS Institute Inc).

We examined whether a child’s sex modified the association between urinary biomarker concentrations and ADHD-related behavior. In secondary analyses, we dichotomized outcomes at the 85th percentile, indicating a possible significant behavior problem. We also considered individual chemicals not included in summary exposure measures (benzophenone-3, triclosan, triclocarban, mono-3-carboxypropyl phthalate [MCPP], monocarboxynonyl phthalate [MCNP], and MHINCH), as well as the individual chemicals that comprised the summary measures.

All models included the child’s sex and mean age at completion of BASC-2 and CADS to adjust for fundamental determinants of behavior, urine-specific gravity to account for urine dilution, and an indicator for ADHD measure. We then used prior literature to inform a directed acyclic graph to select other covariates, including the child’s race/ethnicity (non-Hispanic White vs other) and the following maternal characteristics at the time of delivery: age (as a continuous variable), marital status (married vs unmarried), educational level (<high school vs ≥high school), annual household income (<$20 000 vs ≥$20 000), and smoking during pregnancy (yes or no).^29^

Because not all biomarkers were measured in the first batch of urine samples, some biomarker measures were missing for 27 participants, and some covariate information was missing for 14 participants; thus, our complete case analyses had a sample size of 164 to 190 depending on the urine biomarker measure. In a sensitivity analysis, we imputed missing values (PROC MI/MIANALYZE, Unix SAS, version 9.1.4; SAS Institute Inc) based on 20 imputations using models with all covariates, behavioral outcomes, and biomarker concentrations in this analysis.^30^

We performed additional sensitivity analyses, including adjusting for umbilical cord serum polychlorinated biphenyls and dichlorodiphenyldichloroethylene, and adjusting for 12-month to 36-month maximum blood lead levels, because exposure to these chemicals hase been associated with ADHD-related behaviors in the New Bedford Cohort. We adjusted for whether adolescents had smoked cigarettes in the past 30 days or ever tried alcohol or marijuana (n = 60). We also conducted an unadjusted analysis and analyses separately examining measures reflecting attention vs hyperactivity and impulsivity problems, adjusting for factors associated with increased EDC exposure risk, adjusting for body mass index, adjusting for a family history (parent or sibling) of mental illness (n = 86), adjusting for diagnosis of behavioral problems other than ADHD (n = 74), removing those with a diagnosis of ADHD (n = 39), removing those taking prescription medication for behavioral problems (n = 22), and removing siblings (n = 14). We removed individual BASC-2 behavior measures (12 indices) with instrument validity indicators that suggested a negative or positive bias or lack of internal consistency.

## Results

The mean (SD) age of the 205 participants at assessment was 15.3 (0.7) years, with 112 girls (55%) and 124 non-Hispanic White participants (61%). A substantial proportion of study adolescents were born to mothers who, at the time of delivery, were unmarried (87 [42%]), had less than a high school education (30 [15%]), or had annual household incomes less than $20 000 (66 [32%]) (Table 2 and eTable 1 in the Supplement). Compared with the 528 adolescents participating in the full 15-year follow-up, those in this analysis were younger, and a higher percentage were non-White, but, otherwise, the 2 groups did not differ (eTable 1 in the Supplement).

**Table 2.** Distribution of Characteristics of New Bedford Cohort Adolescents (With EDC Measures) by Parent-Reported ADHD Index on the CADS

Characteristic	Adolescents, No. (%)		
	Without significant or possible behavior problem on CADS ADHD index (n = 148)^a^	Possible significant behavior problem on CADS ADHD index (n = 56)^a^	Significant behavior problem on CADS ADHD index (n = 29)^a^
**Maternal characteristics at time of child’s birth**			
Age, y			
<20	14 (10)	7 (13)	1 (4)
20-29	89 (60)	37 (66)	21 (72)
≥30	45 (30)	12 (21)	7 (24)
Household income, $/y^b^			
<20 000	42 (28)	24 (43)	9 (31)
20 000 to <40 000	47 (32)	23 (41)	17 (59)
40 000 to <75 000	44 (30)	9 (16)	3 (10)
≥75 000	10 (7)	NA	NA
Missing	5 (3)	NA	NA
Educational level^b^			
<High school	12 (8)	18 (32)	10 (34)
≥High school	131 (89)	38 (68)	19 (66)
Missing	5 (3)	NA	NA
Marital status^b^			
Unmarried	53 (36)	34 (61)	14 (48)
Married	88 (59)	18 (32)	12 (41)
Missing	7 (5)	4 (7)	3 (10)
Smoking during pregnancy^b^			
No	109 (74)	25 (45)	12 (41)
Yes	31 (21)	25 (45)	14 (48)
Missing	8 (5)	6 (10)	3 (10)
**Child characteristics**			
Sex			
Male	67 (45)	25 (45)	12 (41)
Female	81 (55)	31 (55)	17 (59)
Mean age at assessment, y			
14-15	118 (80)	45 (80)	23 (79)
16-17	30 (20)	11 (20)	6 (21)
Race^b^			
Non-Hispanic White	99 (67)	25 (45)	16 (55)
Non-White	49 (33)	31 (55)	13 (45)
ADHD diagnosis^b^^,^^c^			
No	137 (93)	28 (50)	12 (41)
Yes	11 (7)	28 (50)	17 (59)
Behavioral problem other than ADHD^b^^,^^c^			
No	107 (72)	23 (41)	11 (38)
Yes	41 (28)	33 (59)	18 (62)
Use of prescription medication for behavioral problem^b^^,^^d^			
No	138 (93)	44 (79)	21 (72)
Yes	10 (7)	12 (21)	8 (28)
Mean number of caffeinated beverages in past 24 h^e^			
0	46 (31)	21 (37)	10 (34)
1	72 (49)	29 (52)	19 (66)
≥2	30 (20)	6 (11)	NA
Personal care product use^e^			
≤6 Products/d	73 (49)	32 (57)	15 (52)
≥7 Products/d	75 (51)	23 (41)	13 (45)
Missing	NA	1 (2)	1 (3)
Fast food consumption^e^			
0 Servings/d	75 (51)	23 (41)	16 (55)
1 Servings/d	65 (44)	27 (48)	11 (38)
>1 Servings/d	8 (5)	5 (9)	1 (3)
Missing	NA	1 (2)	1 (3)
Canned food consumption^e^			
0 Servings/d	114 (77)	49 (87)	23 (79)
≥1 Serving/d	34 (23)	7 (13)	6 (21)
Adolescent substance use^f^			
No	105 (71)	39 (70)	19 (66)
Yes	43 (29)	17 (30)	10 (34)
Peak childhood blood lead level, μg/dL^g^			
<5	57 (38)	19 (34)	10 (34)
≥5	71 (48)	31 (55)	17 (59)
Missing	20 (14)	6 (11)	2 (7)
Body mass index *z* score percentile^h^			
<5th	3 (2)	1 (2)	1 (3)
5th-84th	86 (58)	35 (62)	16 (55)
≥85th	59 (40)	20 (36)	12 (41)

Abbreviations: ADHD, attention-deficit/hyperactivity disorder; CADS, Conners Attention Deficit Scale; EDC, endocrine-disrupting chemical; NA, not applicable.

^a^ Significant behavior problem: scale T-score dichotomized at the 98th percentile; possible significant problem: scale T-score dichotomized at the 85th percentile.

^b^ *P* < .05 for comparisons between those with possible significant behavior problem on CADS ADHD index and those without using the χ^2^ test.

^c^ Medical record or parent-reported diagnosis of a behavioral disorder.

^d^ Parent-reported child use of medication for a behavioral disorder.

^e^ Mean from two 24-hour diary reports (or one 24-hour diary report if only 1 urine sample provided) regarding personal care product use, fast food or canned food consumption, and consumption of caffeinated beverages.

^f^ Adolescent report of having smoked a cigarette in the past 30 days or ever having tried marijuana or alcohol.

^g^ Peak childhood blood lead levels between 12 and 36 months of age.

^h^ Age and sex standardized using Centers for Disease Control and Prevention US reference population.

The median urine concentrations were 0.45 μmol/L of Σantiandrogenic phthalates, 0.13 μmol/L of ΣDEHP metabolites, 0.49 μmol/L of Σpersonal care product phthalates, 0.35 μmol/L of Σparabens, 0.02 μmol/L of Σbisphenols, and 0.02 μmol/L of Σdichlorophenols (Table 1). Individual biomarker concentrations among study participants were similar to those observed in US adolescents in the 2011-2012 National Health and Nutrition Examination Survey.^31^

Of the 205 adolescents in this analysis, 82 (40%) had scores consistent with a significant behavioral problem as defined by at least 1 BASC-2 or CADS ADHD-related measure (Table 3), whereas 39 (19%) had an ADHD diagnosis (Table 2), which is higher than US population estimates (approximately 10%).^1^

**Table 3.** Frequency of ADHD-Related Behavioral Problems in New Bedford Cohort Adolescents as Reported on Teacher-, Parent-, or Self-reported BASC-2 and CADS Behavioral Assessments^a^

Behavioral scale	No. (%)^b^	
	Significant behavior problem^c^	Possible significant behavior problem^d^
BASC-2 teacher-reported inattention	24/173 (14)	55/173 (32)
BASC-2 teacher-reported hyperactivity	16/173 (9)	36/173 (21)
BASC-2 teacher-reported executive function	11/173 (6)	24/173 (14)
BASC-2 parent-reported inattention	13/204 (6)	50/204 (25)
BASC-2 parent-reported hyperactivity	12/204 (6)	31/204 (15)
BASC-2 parent-reported executive function	12/204 (6)	36/204 (18)
BASC-2 self-reported inattention	21/204 (10)	57/204 (28)
BASC-2 self-reported hyperactivity	12/204 (6)	45/204 (22)
CADS teacher-reported ADHD	42/173 (24)	60/173 (35)
CADS teacher-reported inattention	30/173 (17)	43/173 (25)
CADS teacher-reported hyperactivity	35/173 (20)	45/173 (26)
CADS parent-reported ADHD	29/204 (14)	56/204 (27)
CADS parent-reported inattention	20/204 (10)	45/204 (22)
CADS parent-reported hyperactivity	27/204 (13)	45/204 (22)

Abbreviations: ADHD, attention-deficit/hyperactivity disorder; BASC-2, Behavior Assessment System for Children, Second Edition; CADS, Conners Attention Deficit Scale.

^a^ A total of 204 had parent-reported and self-reported scales and 173 had teacher-reported scales.

^b^ A total of 82 had a significant ADHD-associated behavior problem on at least 1 scale and 134 had a possible significant ADHD-associated behavior problem on at least 1 scale.

^c^ Scale T-score dichotomized at the 98th percentile.

^d^ Scale T-score dichotomized at the 85th percentile.

In covariate-adjusted models, summary measures of phthalates were consistently associated with an increased risk of significant ADHD-related behavior problems (Figure; Table 4). A 2-fold increase in Σantiandrogenic phthalates was associated with a 1.34 (95% CI, 1.00-1.79) increased risk of significant ADHD-related behavior problems, whereas a 2-fold increase in ΣDEHP metabolites was associated with a 1.29 (95% CI, 1.07-1.55) increased risk. A smaller 1.16 (95% CI, 0.98-1.37) increased risk was observed with a 2-fold increase in Σpersonal care products. Adverse associations were observed with each individual phthalate metabolite; the strongest association was for MBP (relative risk [RR], 1.45; 95% CI, 1.15-1.84) (eTable 3 in the Supplement). We observed stronger associations for hyperactive behaviors compared with inattentive behaviors for Σantiandrogenic phthalates (RR, 1.40 [95% CI, 1.07-1.84] vs 1.26 [95% CI, 0.87-1.83]), Σpersonal care products (RR, 1.25 [95% CI, 1.07-1.47] vs 1.06 [95% CI, 0.95-1.18]), and Σdichlorophenols (RR, 1.22 [95% CI, 1.04-1.42] vs 1.05 [95% CI, 0.90-1.23]) (Table 4).

**Figure.**
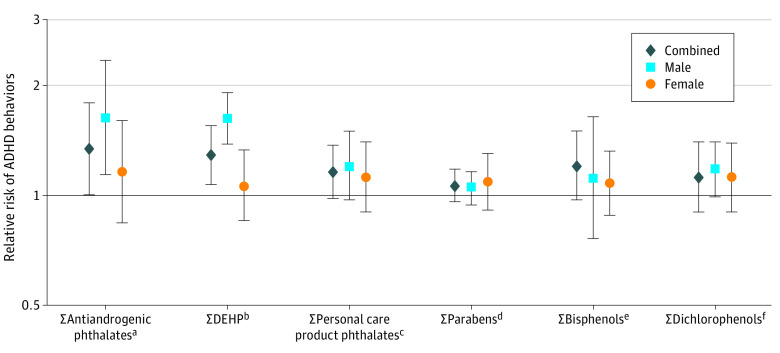
Adjusted Relative Risk of Multiple Measures of Clinically Significant Attention-Deficit/Hyperactivity Disorder (ADHD)–Associated Behaviors Corresponding to a 2-Fold Increase in Urinary Biomarker Concentrations in New Bedford Cohort Adolescents (Sample Size From 164 to 190) Adjusted for child characteristics: sex, race/ethnicity, mean test age (across teacher-reported, parent-reported, and self-reported Behavior Assessment System for Children, 2nd Edition [BASC-2] and Conners Attention Deficit Scale [CADS] measures), and urine specific gravity; maternal characteristics at delivery: age, income, marital status, smoking, and educational level; and test indicator. The ADHD-associated behavior measures include CADS parent-reported and teacher-reported inattention, hyperactivity, and ADHD; BASC-2 parent-reported, teacher-reported, and self-reported hyperactivity and inattention; and BASC-2 parent-reported and teacher-reported executive function. Significant behavior problem: scale T-score dichotomized at the 98th percentile. ^a^Sum of mono-n-butyl phthalate (MBP), monoisobutyl phthalate (MiBP), monobenzyl phthalate, mono-2-ethylhexyl phthalate (MEHP), mono-2-ethyl-5-hydroxyhexyl phthalate (MEHHP), mono-2-ethyl-5-oxohexyl phthalate (MEOHP), mono-2-ethyl-5-carboxypentyl phthalate (MECPP), monocarboxyoctyl phthalate, monohydroxyisobutyl phthalate (MHiBP), monohydroxybutyl phthalate (MHBP), and mono-isononyl phthalate. ^b^Sum of MECPP, MEHHP, MEOHP, and MEHP. ^c^Sum of MBP, MHBP, monoethyl phthalate, MiBP, and MHiBP. ^d^Sum of butyl, ethyl, methyl, and propyl parabens. ^e^Sum of bisphenol A, bisphenol F, and bisphenol S. ^f^Sum of 2,4-dichlorophenol and 2,5-dichlorophenol.

**Table 4.** Adjusted Relative Risk of Multiple Measures of Clinically Significant Behavioral Subgroups of ADHD Behaviors Associated With a 2-Fold Increase in Urinary Biomarker Concentrations in New Bedford Cohort Adolescents (Sample Size From 164 to 190)^a^^,^^b^

Chemical biomarker	Adjusted relative risk		
	Combined ADHD measures	Attention problems^c^	Hyperactivity problems^d^
ΣAntiandrogenic phthalates^e^	1.34 (1.00-1.79)	1.26 (0.87-1.83)	1.40 (1.07-1.84)
ΣDEHP^f^	1.29 (1.07-1.55)	1.29 (1.03-1.60)	1.27 (1.06-1.52)
ΣPersonal care products^g^	1.16 (0.98-1.37)	1.06 (0.85-1.32)	1.25 (1.07-1.47)
ΣParabens^h^	1.06 (0.96-1.18)	1.06 (0.93-1.21)	1.06 (0.95-1.18)
ΣBisphenols^i^	1.09 (0.91-1.31)	1.08 (0.87-1.33)	1.04 (0.84-1.29)
ΣDichlorophenols^j^	1.15 (1.01-1.32)	1.05 (0.90-1.23)	1.22 (1.04-1.42)

Abbreviations: ADHD, attention-deficit/hyperactivity disorder; BASC-2, Behavior Assessment System for Children, 2nd edition; CADS, Conners Attention Deficit Scale; DEHP, di(2-ethylhexyl) phthalate.

^a^ Adjusted for child: sex, mean test age (across teacher-reported, parent-reported, and self-reported BASC and CADS measures), and urine specific gravity; maternal characteristics at delivery: age, income, marital status, smoking, and educational level; and test indicator.

^b^ Attention-deficit/hyperactivity disorder–related behavior measures include CADS parent-reported and teacher-reported inattention, hyperactivity, and ADHD; BASC-2 parent-reported, teacher-reported, and self-reported hyperactivity and inattention; and BASC-2 parent-reported and teacher-reported executive function. Significant behavior problem: scale T-score dichotomized at the 98th percentile.

^c^ Attention problems subset includes CADS teacher-reported and parent-reported inattention and BASC-2 teacher-reported, parent-reported, and self-reported inattention.

^d^ Hyperactivity subset includes CADS teacher-reported and parent-reported hyperactive behavior and BASC-2 teacher-reported, parent-reported, and self-reported hyperactive behavior.

^e^ Sum of MBP, MiBP, MBzP, MEHP, MEHHP, MEOHP, MECPP, MCOP, MHiBP, MHBP, and MNP (full chemical names in Table 1).

^f^ Sum of MECPP, MEHHP, MEOHP, and MEHP (full chemical names in Table 1).

^g^ Sum of MBP, MHBP, MEP, MiBP, and MHiBP (full chemical names in Table 1).

^h^ Sum of butyl, ethyl, methyl, and propyl parabens.

^i^ Sum of BPA, BPF, and BPS (full chemical names in Table 1).

^j^ Sum of 2,4-dichlorophenol and 2,5-dichlorophenol.

Associations tended to be stronger among male adolescents than among female adolescents, most notably with respect to the ΣDEHP metabolites (*P* = .002 for interaction), in which a 2-fold increase corresponded to a 1.62 (95% CI, 1.38-1.91) increased risk of significant ADHD-related behavior problems in male adolescents compared with a 1.06 (95% CI, −1.15 to 1.33) increased risk in female adolescents (Figure; eTable 2 in the Supplement). The remaining sex-specific differences were estimated with less precision.

A 2-fold increase in Σdichlorophenols was also associated with an increased risk of ADHD-related behaviors (RR, 1.15; 95% CI, 1.01-1.32) (Table 4). However, we did not observe associations with urinary biomarker concentrations of Σparabens or Σbisphenols or with the individual components of Σparabens or Σbisphenols summary measures, triclocarban, triclosan, benzophenone-3, or MHINCH (Figure; eTables 2 and 3 in the Supplement).

When considering the more inclusive threshold for those with possible significant behavioral problems (>85th percentile), we observed similar, albeit slightly attenuated, findings. For example, each 2-fold increase in Σantiandrogenic phthalates was associated with a 1.21 (95% CI, 1.00-1.47) increased risk of possible significant (vs 1.34 for clearly significant) ADHD-related behavior problems (Table 4), and this difference appeared to be more pronounced among male adolescents (eTable 2 in the Supplement).

Associations were similar, but more precise, in models imputing missing data (eTable 4 in the Supplement). Associations were stronger for summary phthalate measures when we excluded adolescents with an ADHD diagnosis. We did not observe substantive changes in results with the other sensitivity analyses, including exclusion of those taking medications (eTables 4 and 5 in the Supplement).

## Discussion

In a population with a high prevalence of ADHD-related behaviors, our findings support an association of adolescent exposure to EDCs, particularly select phthalates, with an increased risk of significant ADHD-related behavior problems at exposure biomarker concentrations typical of adolescents in the general US population. These results are consistent with previously reported findings in the New Bedford Cohort associating adolescent EDC exposure, particularly to antiandrogenic phthalates, with increased externalizing behaviors (a BASC-2 composite of hyperactivity, aggression, and conduct problems).^16^

Previous studies have reported associations of prenatal and early postnatal exposure to phthalates and BPA with an increase in ADHD-related behaviors. However, to our knowledge, no other published studies have analyzed associations between adolescent EDC biomarker concentrations and ADHD-related behaviors. Adolescence is a critical period for brain development and may be another time of heightened vulnerability to EDC exposure. Several cross-sectional studies (with children of wide age ranges, including some adolescents) have reported associations between increased urinary phthalate and BPA concentrations and ADHD-related behaviors.^5,7,9,32^ Experimental animal studies also support the potential for EDC exposures to be associated with adverse behavior in adolescence, including behaviors consistent with ADHD.^33,34,35,36^ Furthermore, there is well-established evidence demonstrating the unique association that neurotoxicant exposures (eg, alcohol and organophosphates) during adolescence have with adverse behavior.^37,38^

This analysis focused on ADHD-related behaviors as opposed to clinical ADHD diagnosis. By relying on multiple indices of ADHD-related behavior (from the BASC-2 and CADS) from multiple observers (teachers, parents, and self), we were able to leverage a uniquely comprehensive characterization of adolescent behavior, including behaviors that can vary over time (eg, inattention) and thus may be susceptible to variations in exposure risk factors. In particular, combining information from these multiple measures increased the power of our analysis, resulting in more stable estimates of association than would have been possible by examining each behavioral measure individually. In addition, by expressing multiple measures as a single outcome, we did not induce the statistical issues associated with multiple comparisons.

### Limitations

This study has some limitations. The assessed EDCs have short elimination half-lives, as well as short-term variability in exposure, resulting in the potential for nondifferential exposure misclassification, which could bias results toward the null. However, our study collected urine samples at 2 different times from most participants, allowing us to better characterize each participant’s mean or usual exposure. Given the cross-sectional design of this analysis, another challenge is the potential for reverse causation, such that existing behavioral problems may alter habits that enhance EDC exposure via changes in, for instance, diet, substance use, and/or personal care product use.^39,40,41,42^ However, adjustments for these factors did not affect associations, suggesting that reverse causation from these habits was unlikely. Furthermore, excluding adolescents with a diagnosis of ADHD enhanced the existing associations (eTable 4 in the Supplement). Although data on participants’ prenatal EDC exposure are lacking, we do not expect this lack of data would confound associations with adolescent exposure; weak within-person correlations have been reported between prenatal and childhood exposures to phthalates and BPA, and we would expect similar or weaker correlations between prenatal and adolescent time periods.^43,44^ In addition, participants were from a small geographical region, which may potentially affect the generalizability of our findings.

## Conclusions

Our results support the importance of adolescent exposure to EDCs, particularly phthalates, as a potential risk factor for significant ADHD-related behavior problems. Attention-deficit/hyperactivity disorder is a common and costly neurobehavioral disorder; US health care and education expenditures associated with ADHD in children and adolescents are estimated to be $38 billion to $72 billion annually, while costs associated with ADHD in adults, including lost wages, are much higher, ranging from $143 billion to $266 billion annually.^2,45^ The identification of modifiable risk factors for ADHD is of great public health importance. These findings contribute new insights into the potential detrimental neurobehavioral outcomes of EDC exposure during adolescence.

## Notes

Supplement. **eTable 1.** Characteristics of 528 New Bedford Cohort Participants Assessed as Adolescents Compared With the Subset of 205 Who Provided Urine Samples for EDC Exposure Assessment **eTable 2.** Adjusted Relative Risk (95% Confidence Intervals) of ADHD-Related Behavior Problems Associated With a 2-Fold (log2) Increase in Urinary Biomarker Concentrations in New Bedford Cohort Adolescents (N = 164-190) **eTable 3.** Adjusted Relative Risk (95% Confidence Interval) of Significant ADHD-Related Behavior Problems in New Bedford Cohort Adolescents Associated With a 2-Fold (log2) Increase in Urinary Biomarker Concentrations (N = 164-190) for Individual, Rather Than Summed, Chemical Measures **eTable 4.** Sensitivity Analyses for the Adjusted Relative Risk (95% Confidence Interval) of Significant ADHD-Related Behavior Problems Associated With a 2-Fold (log2) Increase in Urinary Biomarker Concentrations in New Bedford Cohort Adolescents **eTable 5.** Additional Sensitivity Analyses for the Adjusted Relative Risk (95% Confidence Interval) of Significant ADHD-Related Behavior Problems Associated With a 2-Fold (log2) Increase in Urinary Biomarker Concentrations in New Bedford Cohort Adolescents **eFigure.** Determination of Final Analytic Sample for Assessment of New Bedford Cohort Adolescents
