# Supplementary material for: Association of Exposure to Endocrine-Disrupting Chemicals During Adolescence With Attention-Deficit/Hyperactivity Disorder–Related Behaviors
Source: JAMA Netw Open. 2020 Aug 28;3(8):e2015041. doi: 10.1001/jamanetworkopen.2020.15041 (PMC7455852; doi:10.1001/jamanetworkopen.2020.15041)
Supplement: Supplement. — eTable 1. Characteristics of 528 New Bedford Cohort Participants Assessed as Adolescents Compared With the Subset of 205 Who Provided Urine Samples for EDC Exposure Assessment eTable 2. Adjusted Relative Risk (95% Confidence Intervals) of ADHD-Related Behavior Problems Associated With a 2-Fold (log2) Increase in Urinary Biomarker Concentrations in New Bedford Cohort Adolescents (N = 164-190) eTable 3. Adjusted Relative Risk (95% Confidence Interval) of Significant ADHD-Related Behavior Problems in New Bedford Cohort Adolescents Associated With a 2-Fold (log2) Increase in Urinary Biomarker Concentrations (N = 164-190) for Individual, Rather Than Summed, Chemical Measures eTable 4. Sensitivity Analyses for the Adjusted Relative Risk (95% Confidence Interval) of Significant ADHD-Related Behavior Problems Associated With a 2-Fold (log2) Increase in Urinary Biomarker Concentrations in New Bedford Cohort Adolescents eTable 5. Additional Sensitivity Analyses for the Adjusted Relative Risk (95% Confidence Interval) of Significant ADHD-Related Behavior Problems Associated With a 2-Fold (log2) Increase in Urinary Biomarker Concentrations in New Bedford Cohort Adolescents eFigure. Determination of Final Analytic Sample for Assessment of New Bedford Cohort Adolescents [file jamanetwopen-3-e2015041-s001.pdf]

Supplementary Online Content

Shoaff JR, Coull B, Weuve J, et al. Association of exposure to endocrine-disrupting chemicals during adolescence with attention-deficit/hyperactivity disorder–related behaviors. *JAMA Netw Open.* 2020;3(8):e2015041.
doi:10.1001/jamanetworkopen.2020.15041

eTable 1. Characteristics of 528 New Bedford Cohort Participants Assessed as Adolescents Compared With the Subset of 205 Who Provided Urine Samples for EDC Exposure Assessment
eTable 2. Adjusted Relative Risk (95% Confidence Intervals) of ADHD-Related Behavior Problems Associated With a 2-Fold (log2) Increase in Urinary Biomarker Concentrations in New Bedford Cohort Adolescents (N=164-190)
eTable 3. Adjusted Relative Risk (95% Confidence Interval) of Significant ADHD-Related Behavior Problems in New Bedford Cohort Adolescents Associated With a 2-Fold (log2) Increase in Urinary Biomarker Concentrations (N=164-190) for Individual, Rather Than Summed, Chemical Measures
eTable 4. Sensitivity Analyses for the Adjusted Relative Risk (95% Confidence Interval) of Significant ADHD-Related Behavior Problems Associated With a 2-Fold (log2) Increase in Urinary Biomarker Concentrations in New Bedford Cohort Adolescents
eTable 5. Additional Sensitivity Analyses for the Adjusted Relative Risk (95% Confidence Interval) of Significant ADHD-Related Behavior Problems Associated With a 2-Fold (log2) Increase in Urinary Biomarker Concentrations in New Bedford Cohort Adolescents
eFigure. Determination of Final Analytic Sample for Assessment of New Bedford Cohort Adolescents

This supplementary material has been provided by the authors to give readers additional information about their work.

© 2020 Shoaff JR et al. *JAMA Network Open.*

eTable 1. Characteristics of 528 New Bedford Cohort Participants Assessed as Adolescents Compared With the Subset of 205 Who Provided Urine Samples for EDC Exposure Assessment

	Urine study sample (n=205)	Full sample (n=528)
**Frequency of ADHD-related behavioral problems (n, (%))[a]**		
Significant ADHD-associated behavior problem on at least one scale[b]	82 (40)	218 (41)
Possible significant ADHD-associated behavior problem on at least one scale[b]	134 (65)	346 (65)
**Child Characteristics (n (%))**		
**Gender**		
Male	93 (45)	255 (48)
Female	112 (55)	273 (52)
Missing	0 (0)	0 (0)
**Race**		
Non-Hispanic White	124 (60)	364 (69)
Non White	81 (40)	164 (31)
Missing	0 (0)	0 (0)
**Age at in-person testing**		
14-15	164 (80)	328 (62)
16-17	41 (20)	200 (38)
Missing		
**Diagnosis of a behavioral disorder other than ADHD[c]**		
No	131 (64)	346 (66)
Yes	74 (36)	182 (34)
Missing	0 (0)	0 (0)
**Use of medication for a behavioral disorder[d]**		
No	183 (89)	480 (91)
Yes	22 (11)	48 (9)
Missing	0 (0)	0 (0)
**Maternal Characteristics at time of Child's Birth (n (%))**		
**Age (years)**		
<20	21 (10)	67 (13)
20-29	127 (62)	307 (58)
≥30	57 (28)	154 (29)
Missing	0 (0)	0 (0)
**Household income ($/year)**		
<20,000	66 (32)	166 (31)
20-<40,000	70 (34)	179 (34)
40-<75,000	53 (26)	152 (29)
≥75,000	10 (5)	18 (3)
Missing	6 (3)	13 (2)
**Marital status**		

© 2020 Shoaff JR et al. *JAMA Network Open.*

Married	106 (52)	296 (56)
Unmarried	87 (42)	206 (39)
Missing	12 (6)	26 (5)
**Education**		
<High School	30 (15)	76 (14)
High School or more	169 (82)	440 (83)
Missing	6 (3)	12 (2)
**Smoking during pregnancy**		
Yes	56 (27)	125 (24)
No	134 (65)	362 (69)
Missing	15 (7)	41 (8)
**Alcohol consumption during pregnancy**		
Yes	38 (19)	92 (17)
No	141 (69)	358 (68)
Missing	26 (13)	78 (15)

[a]ADHD-related behavior measures include: CADS parent/teacher-report of inattention, hyperactivity, and ADHD; BASC parent/teacher/self-report of hyperactivity and inattention; and BASC parent/teacher-report of executive function.
[b]Significant behavior problem: scale t-score dichotomized at the 98th percentile; Possible significant problem: scale t-score dichotomized at the 85th percentile
[c]Pediatric medical record or parental reported diagnosis of a behavioral disorder
[d]Parental reported child use of medication for a behavioral disorder

© 2020 Shoaff JR et al. *JAMA Network Open.*

eTable 2. Adjusted[a] Relative Risk (95% Confidence Intervals) of ADHD-Related Behavior Problems[b] Associated With a 2-Fold (log2) Increase in Urinary Biomarker Concentrations in New Bedford Cohort Adolescents (N=164-190)

Chemical Biomarker	Significant behavior problem[b]			Possible significant behavior problem[b]		
	Combined	Males	Females	Combined	Males	Females
ΣAntiandrogenic phthalates[c]	1.34 (1.00, 1.79)	1.63 (1.14, 2.34)	1.16 (0.84, 1.60)	1.21 (1.00, 1.47)	1.19 (0.87, 1.63)	1.23 (0.99, 1.52)
ΣDEHP[d]	1.29 (1.07, 1.55)	1.62 (1.38, 1.91)*	1.06 (0.85, 1.33)*	1.20 (1.05, 1.37)	1.28 (1.06, 1.55)	1.14 (0.98, 1.32)
ΣPersonal care products[e]	1.16 (0.98, 1.37)	1.20 (0.97, 1.50)	1.12 (0.90, 1.40)	1.11 (0.99, 1.25)	1.08 (0.90, 1.30)	1.14 (0.98, 1.32)
ΣParabens[f]	1.06 (0.96, 1.18)	1.05 (0.94, 1.16)	1.09 (0.91, 1.30)	1.04 (0.97, 1.11)	1.01 (0.93, 1.09)	1.08 (0.96, 1.21)
ΣBisphenols[g]	1.09 (0.91, 1.31)	1.11 (0.76, 1.64)	1.08 (0.88, 1.32)	1.01 (0.88, 1.17)	0.92 (0.70, 1.22)	1.06 (0.89, 1.26)
ΣDichlorophenols[h]	1.15 (1.01, 1.32)	1.18 (0.99, 1.40)	1.12 (0.90, 1.39)	1.09 (0.99, 1.20)	1.07 (0.94, 1.21)	1.13 (0.97, 1.33)

[a]Adjusted for: Child: sex, race, mean test age (across teacher/parent/self-report BASC and CADS measures), urine specific gravity, race; Maternal characteristics at delivery: age, income, marital status, smoking, education; test indicator
[b]ADHD-related behavior measures include: CADS parent/teacher-report of inattention, hyperactivity, and ADHD; BASC parent/teacher/self-report of hyperactivity and inattention; and BASC parent/teacher-report of executive function. Significant behavior problem: scale t-score dichotomized at the 98th percentile; Possible significant problem: scale t-score dichotomized at the 85th percentile.
[c]sum of: MBP, MiBP, MBzP, MEHP, MEHHP, MEOHP, MECPP, MCOP, MHIBP, MHBP, MNP
[d]sum of: MECPP, MEHHP, MEOHP, MEHP
[e]sum of: MBP, MHBP, MEP, MiBP, MHIBP
[f]sum of: butyl, ethyl, methyl, and propyl parabens
[g]sum of: BPA, BPF, BPS
[h]sum of: 2,4-dichlorophenol, 2,5-dichlorophenol
*Interaction p-value <0.05

© 2020 Shoaff JR et al. *JAMA Network Open.*

eTable 3. Adjusted[a] Relative Risk (95% Confidence Interval) of Significant ADHD-Related Behavior Problems[b] in New Bedford Cohort Adolescents Associated With a 2-Fold (log2) Increase in Urinary Biomarker Concentrations (N=164-190) for Individual, Rather Than Summed, Chemical Measures

ΣAntiandrogenic phthalates		ΣDEHP		ΣPersonal care products		ΣParabens		ΣBisphenols		ΣDichlorophenols		Individual measures not included in a sum	
MECPP	1.27 (1.05, 1.54)	MECPP	1.27 (1.05, 1.54)	MBP	1.45 (1.15, 1.84)	Butyl	0.98 (0.95, 1.02)	BPA	1.02 (0.93, 1.12)	2,4-DCP	1.11 (1.00, 1.24)	TCC	1.03 (1.00, 1.07)
MEHHP	1.26 (1.06, 1.49)	MEHHP	1.26 (1.06, 1.49)	MIBP	1.32 (1.07, 1.64)	Ethyl	1.07 (0.98, 1.17)	BPF	1.01 (0.97, 1.05)	2,5-DCP	1.08 (0.94, 1.25)	TCS	0.99 (0.89, 1.09)
MEHP	1.05 (0.98, 1.12)	MEHP	1.05 (0.98, 1.12)	MHIBP	1.16 (0.96, 1.41)	Methyl	1.05 (0.94, 1.16)	BPS	1.02 (0.94, 1.10)			BP-3	0.97 (0.9, 1.04)
MEOHP	1.28 (1.07, 1.53)	MEOHP	1.28 (1.07, 1.53)	MHBP	1.25 (1.03, 1.51)	Propyl	1.00 (0.92, 1.09)					MHINCH	0.99 (0.94, 1.05)
MBP	1.45 (1.15, 1.84)			MEP	1.07 (0.93, 1.23)							MCNP	1.19 (0.95, 1.49)
MIBP	1.32 (1.07, 1.64)											MCPP	1.12 (1.02, 1.23)
MHIBP	1.16 (0.96, 1.41)												
MHBP	1.25 (1.03, 1.51)												
MBZP	1.22 (1.05, 1.42)												
MNP	1.03 (0.97, 1.10)												
MCOP	1.05 (0.91, 1.23)												

[a]Adjusted for: Child: sex, race, mean test age (across teacher/parent/self-report BASC and CADS measures), urine specific gravity; Maternal characteristics at delivery: age, income, marital status, smoking, education; test indicator

[b]ADHD-related behavior measures include: CADS parent/teacher-report of inattention, hyperactivity, and ADHD; BASC parent/teacher/self-report of hyperactivity and inattention; and BASC parent/teacher-report of executive function. Significant behavior problem: scale t-score dichotomized at the 98th percentile

© 2020 Shoaff JR et al. *JAMA Network Open.*

eTable 4. Sensitivity Analyses for the Adjusted[a] Relative Risk (95% Confidence Interval) of Significant ADHD-Related Behavior Problems[b] Associated With a 2-Fold (log2) Increase in Urinary Biomarker Concentrations in New Bedford Cohort Adolescents

Chemical Biomarker	Main adjusted model[a] N=164-190	Unadjusted model[c] N=178-205	Multiple imputation[d] N=205	Exclude BASC measures with indices indicating a potential threat to validity[e] N=164-190	Exclude participants taking medications for behavioral disorder[f] N=149-171	Exclude participants with ADHD diagnosis[g] N=135-156	Adjusted for behavioral diagnosis other than ADHD[g] N=164-190	Adjusted for family history of mental illness[h] N=164-190	Exclude siblings N=153-177
ΣAntiandrogenic phthalates[i]	1.34 (1.00, 1.79)	1.27 (1.03, 1.56)	1.37 (1.21, 1.55)	1.34 (1.01, 1.78)	1.26 (0.90, 1.78)	1.76 (1.33, 2.32)	1.34 (1.01, 1.78)	1.40 (1.03, 1.89)	1.33 (0.99, 1.79)
ΣDEHP[j]	1.29 (1.07, 1.55)	1.19 (1.02, 1.39)	1.25 (1.14, 1.38)	1.28 (1.06, 1.54)	1.32 (1.07, 1.62)	1.46 (1.19, 1.80)	1.30 (1.09, 1.56)	1.30 (1.08, 1.58)	1.29 (1.07, 1.55)
ΣPersonal care products[k]	1.16 (0.98, 1.37)	1.20 (1.03, 1.39)	1.15 (1.05, 1.25)	1.16 (0.98, 1.37)	1.11 (0.90, 1.37)	1.31 (1.09, 1.57)	1.15 (0.97, 1.36)	1.18 (1.00, 1.40)	1.18 (0.99, 1.41)
ΣParabens[l]	1.06 (0.96, 1.18)	1.06 (0.96, 1.17)	1.03 (0.98, 1.08)	1.05 (0.95, 1.17)	1.07 (0.95, 1.20)	1.09 (0.99, 1.20)	1.06 (0.96, 1.17)	1.09 (0.98, 1.21)	1.07 (0.96, 1.18)
ΣBisphenols[m]	1.09 (0.91, 1.31)	1.09 (0.91, 1.31)	1.10 (0.99, 1.23)	1.06 (0.87, 1.27)	1.12 (0.92, 1.36)	1.13 (0.88, 1.45)	1.10 (0.91, 1.33)	1.12 (0.94, 1.33)	1.08 (0.91, 1.29)
ΣDichlorophenols[n]	1.15 (1.01, 1.32)	1.09 (0.94, 1.26)	1.11 (1.03, 1.20)	1.15 (1.01, 1.31)	1.12 (0.96, 1.32)	1.15 (1.00, 1.33)	1.17 (1.02, 1.34)	1.15 (1.00, 1.33)	1.18 (1.02, 1.35)

[a]Adjusted for: Child: sex, race, mean test age (across teacher/parent/self-report BASC and CADS measures), urine specific gravity; Maternal characteristics at delivery: age, income, marital status, smoking, education; test indicator

[b]ADHD-related behavior measures include: CADS parent/teacher-report of inattention, hyperactivity, and ADHD; BASC parent/teacher/self-report of hyperactivity and inattention; and BASC parent/teacher-report of executive function. Significant behavior problem: scale t-score dichotomized at the 98th percentile.

[c]Adjusted for specific gravity to account for urine dilution and test indicator

[d]imputation for covariates and chemicals, but not ADHD-related measures

[e]Internal BASC indices indicating potential threats to validity from several factors (overly negative, lack of internal consistency, etc).

[f]Based on medical record review

[g]Based on medical record review and parent report

[h]Based on parent report

[i]sum of: MBP, MiBP, MBzP, MEHP, MEHHP, MEOHP, MECPP, MCOP, MHIBP, MHBP, MNP

[j]sum of: MECPP, MEHHP, MEOHP, MEHP

[k]sum of: MBP, MHBP, MEP, MiBP, MHIBP

[l]sum of: butyl, ethyl, methyl, and propyl parabens

[m]sum of: BPA, BPF, BPS

[n]sum of: 2,4-dichlorophenol, 2,5-dichlorophenol

© 2020 Shoaff JR et al. *JAMA Network Open.*

eTable 5. Additional Sensitivity Analyses for the Adjusted[a] Relative Risk (95% Confidence Interval) of Significant ADHD-Related Behavior Problems[b] Associated With a 2-Fold (log2) Increase in Urinary Biomarker Concentrations in New Bedford Cohort Adolescents

Chemical Biomarker	Main adjusted model N=164-190	Adjusted for adolescent BMI z-score N=164-190	Adjusted for max childhood blood lead N=147-189	Adjusted for cord serum PCBs and DDE N=163-189	Adjusted for adolescent substance use[c] N=164-190	Adjusted for caffeine consumption[d] N=164-190	Adjusted for personal care product use[d] N=163-189	Adjusted for canned food and fast food consumption[d] N=163-189
ΣAntiandrogenic phthalates[f]	1.34 (1.00, 1.79)	1.33 (0.99, 1.78)	1.30 (0.97, 1.76)	1.36 (1.01, 1.83)	1.27 (0.96, 1.68)	1.34 (1.01, 1.78)	1.37 (1.02, 1.85)	1.36 (1.00, 1.85)
ΣDEHP[g]	1.29 (1.07, 1.55)	1.29 (1.07, 1.55)	1.26 (1.05, 1.52)	1.29 (1.06, 1.57)	1.25 (1.03, 1.51)	1.30 (1.09, 1.56)	1.29 (1.08, 1.55)	1.31 (1.08, 1.59)
ΣPersonal care products[h]	1.16 (0.98, 1.37)	1.16 (0.97, 1.38)	1.14 (0.94, 1.38)	1.16 (0.98, 1.38)	1.15 (0.95, 1.38)	1.15 (0.97, 1.36)	1.16 (0.98, 1.38)	1.17 (0.98, 1.39)
ΣParabens[i]	1.06 (0.96, 1.18)	1.07 (0.96, 1.18)	1.07 (0.96, 1.19)	1.04 (0.94, 1.15)	1.04 (0.94, 1.14)	1.06 (0.96, 1.17)	1.07 (0.97, 1.18)	1.07 (0.96, 1.18)
ΣBisphenols[j]	1.09 (0.91, 1.31)	1.09 (0.90, 1.30)	1.05 (0.87, 1.27)	1.08 (0.90, 1.29)	1.09 (0.90, 1.33)	1.10 (0.91, 1.33)	1.08 (0.90, 1.31)	1.07 (0.88, 1.31)
ΣDichlorophenols[k]	1.15 (1.01,1.32)	1.15 (1.01, 1.32)	1.19 (1.01, 1.40)	1.17 (1.02, 1.34)	1.13 (0.98, 1.31)	1.17 (1.02, 1.34)	1.16 (1.01, 1.33)	1.16 (1.01, 1.33)

[a]Adjusted for: Child: sex, mean test age (across teacher/parent/self-report BASC and CADS measures), urine specific gravity; Maternal characteristics at delivery: age, income, marital status, smoking, education; test indicator

[b]ADHD-related behavior measures include: CADS parent/teacher-report of inattention, hyperactivity, and ADHD; BASC parent/teacher/self-report of hyperactivity and inattention; and BASC parent/teacher-report of executive function. Significant behavior problem: scale t-score dichotomized at the 98th percentile

[c]Adolescent report of having smoked a cigarette in the past 30 days or ever having tried marijuana or alcohol

[d]Average from two 24-hour diary reports (or one 24-hour diary report if only one urine sample provided) regarding personal care product use, canned/fast food consumption, and consumption of caffeinated beverage

[f]sum of: MBP, MiBP, MBzP, MEHP, MEHHP, MEOHP, MECPP, MCOP, MHIBP, MHBP, MNP

[g]sum of: MECPP, MEHHP, MEOHP, MEHP

[h]sum of: MBP, MHBP, MEP, MiBP, MHIBP

[i]sum of: butyl, ethyl, methyl, and propyl parabens

[j]sum of: BPA, BPF, BPS

[k]sum of: 2,4-dichlorophenol, 2,5-dichlorophenol

© 2020 Shoaff JR et al. *JAMA Network Open.*

eFigure 1. Determination of final analytic sample for assessment of assessment of New Bedford Cohort adolescents

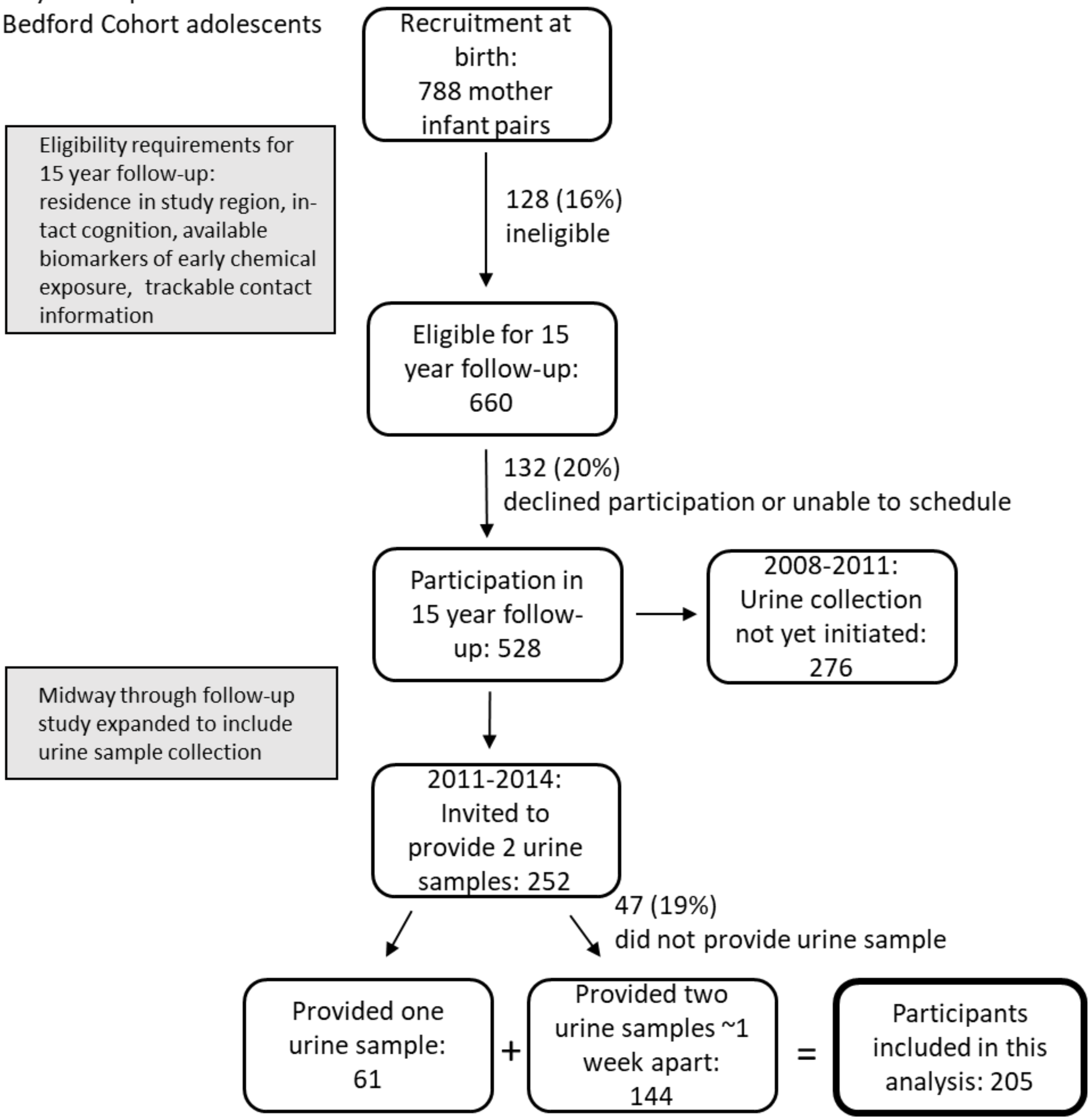

© 2020 Shoaff JR et al. *JAMA Network Open.*
